# Using probiotics to improve nutrient digestibility and gut-health of weaned pigs: a comparison of maternal and nursery supplementation strategies

**DOI:** 10.3389/fvets.2024.1356455

**Published:** 2024-03-22

**Authors:** Gabriela Miotto Galli, Ines Andretta, Crystal Levesque, Thais Stefanello, Camila Lopes Carvalho, Jorge Yair Perez Pelencia, Gabriel Bueno Martins, Bruna Souza de Lima Cony, Caroline Romeiro de Oliveira, Carolina Haubert Franceschi, Marcos Kipper

**Affiliations:** ^1^Department of Animal Science, Universidade Federal do Rio Grande do Sul, Porto Alegre, Rio Grande do Sul, Brazil; ^2^Department of Animal Science, South Dakota State University, Brookings, SD, United States; ^3^Elanco Animal Health, São Paulo, Brazil

**Keywords:** intestinal health, nursery, probiotic, serum biochemistry, sow, piglet

## Abstract

Maternal probiotic supplementation has been found to have a positive impact on the gut health of piglets, not only during the lactation period, but also after weaning. Providing probiotics to nursery pigs is also a common strategy for supplementation. The goal of this study was to evaluate which would be the most effective strategy to improve nutrient digestibility, energy metabolism, and intestinal health in weaned pigs considering the maternal or nursery options. A total of 32 newly weaned pigs were randomly split into a 2 × 2 factorial arrangement considering maternal probiotic supplementation (with or without) in gestation-lactation and probiotic supplementation in the nursery period (with or without). After weaning, experimental diets were provided for 22 days. Total fecal and urine collection was performed from day 15 to 21. Blood samples were collected from all pigs on days 3 and 22 of the experiment to assess serum biochemistry and intestinal permeability. All pigs were euthanized on day 22 for intestinal tissue collection. Pigs born from probiotic-fed sows had greater (*p* < 0.05) total tract digestibility of dry matter (+1%) and gross energy (+1.3%), and greater (*p* < 0.05) metabolizable energy coefficient (+1.3%), which resulted in a 46 kcal/kg increase (*p* < 0.05) in the metabolizable energy content of the diet. Nitrogen intake (*p* = 0.035), uptake (*p* = 0.007), and retention (*p* = 0.012) were all increased in these pigs. Fecal moisture was reduced in pigs born from probiotic-fed sows and pigs fed the probiotic diet only in the nursery (*p* < 0.05). Pigs born from probiotic-fed sows had reduced intestinal permeability by 16% (*p* < 0.05), whereas pigs fed the probiotic diet in the nursery only tended to improve this response (*p* < 0.10). The villus:crypt ratio of pigs born from probiotic-fed sows was greater compared to the control (*p* < 0.05), while serum levels of alanine aminotransferase were lower (*p* < 0.05). Pigs born from probiotic-fed sows had increased nutrient digestibility and improved gut health. Therefore, it is concluded that supplementing the sow diets with probiotics rather than just providing diets in the nursery phase is an advantageous strategy.

## 1 Introduction

Weaning is a crucial stage for piglets, as it has the potential to significantly affect their intestinal functions. The weaning stress is also closely related to social challenges (e.g., separation from the sow, and hierarchy formation) and crucial alterations in feeding behavior (e.g., a transition from liquid to solid diet), that eventually can result in suboptimal performance. Thus, pre-weaning strategies that can optimize piglet physiological preparation are crucial for improving post-weaning pig performance and health status, especially in the context of reduced use of feed antibiotics (1).

One of these pre-weaning strategies to alleviate the adverse consequences of weaning is the maternal supplementation with probiotics, which are additives composed by live microorganisms that may confer a health benefit on the host (2). Probiotics can improve intestinal morphology and barrier function (3), increase enzyme activity (4), increase the production of short-chain volatile fatty acids (5), and reduce diarrhea, which can lead to an improvement in growth performance (5).

Although several beneficial effects of probiotics have already been reported in the post-weaning phase, it is crucial to remember that pig performance in the nursery is closely related to what happens in the previous suckling phase (6). Furthermore, the conditions that the mother experiences throughout her pregnancy have an impact on the growth and development of her offspring throughout their lives (7). Numerous mechanisms have already been proposed to explain this impact. For instance, the neonatal gastrointestinal tract is colonized by bacteria acquired from the sow during farrowing and throughout lactation (6). Previous studies revealed that nursing piglets born from probiotic-fed sows carried over some modification in fecal microbial population that happened preweaning (8). Thus, probiotic supplementation to the sows during gestation and lactation may modulate the offspring immunity and gut health (9), even during the nursery phase.

Although the maternal administration of probiotics is a viable approach, providing probiotics directly to weaned piglets is generally a more commonly used strategy of supplementation. Only few studies comparing these supplementation strategies are available, particularly when it comes to metabolic and gut-health responses. Therefore, the goal of the present research was to evaluate probiotic supplementation provided to sows or directly to the weaned pigs in the nursery phase in order to access which would be the best strategy to improve nutrient digestibility, energy metabolism, and intestinal health in weaned pigs.

## 2 Materials and methods

### 2.1 Treatments

A factorial 2 × 2 design was used, considering probiotic supplementation during gestation-lactation (control or supplemented) and during the nursery period (control or supplemented). The tested feed additive (Protexin™ Concentrate, Elanco Animal Health, São Paulo, Brazil) contained *Lactobacillus acidophilus* (2.06×10^8^ CFU/g), *Lactobacillus bulgaricus* (2.06×10^8^ CFU/g), *Lactobacillus plantarum* (1.26×10^8^ CFU/g), *Lactobacillus rhamnosus* (2.06×10^8^ CFU/g), *Bifidobacterium bifidum* (2.0×10^8^ CFU/g), *Enterococcus faecium* (6.46×10^8^ CFU/g), and *Streptococcus thermophilus* (4.10×10^8^ CFU/g). The same additive was used for gestating-lactating sows (50 g of probiotic additive per ton of feed) and during the nursery phase (200 g of additive per ton of feed).

### 2.2 Gestation-lactation phase

The gestation-lactation phase of the experiment was developed on a commercial farm located in Maratá (Rio Grande do Sul, Southern Region, Brazil). Two hundred sows (Camborough®, PIC, São Paulo, Brazil) with parity orders ranging from 2 to 9 do were assigned (randomly within each parity order) to the treatments. Supplementation started on the first day of pregnancy and persisted until the end of lactation. To prevent cross-contamination among sows of different treatments, the probiotics were provided using gelatin capsules directly into the mouth of each sow every day according to the treatment. The daily individual supplementation was calculated considering an average feed intake for the period based on historical data from the barn (i.e., 1.8 kg/day during gestation and 7 kg/day during lactation) disregarding among-animal variation.

Feeding programs during gestation considered the body condition of the sow to provide from 1.5 to 1.9 kg of corn/soybean meal-based feed once a day for each animal. Lactating sows were fed *ad libitum* five times a day. In both phases, feeding programs followed the recommendations of the genetic company (PIC nutrition and feeding guidelines) and are representatives of the standard programs in Brazilian pig production. Creep feed was not provided to the piglets. Water was supplied from individual nipple drinkers to all the piglets.

**Figure 1:**
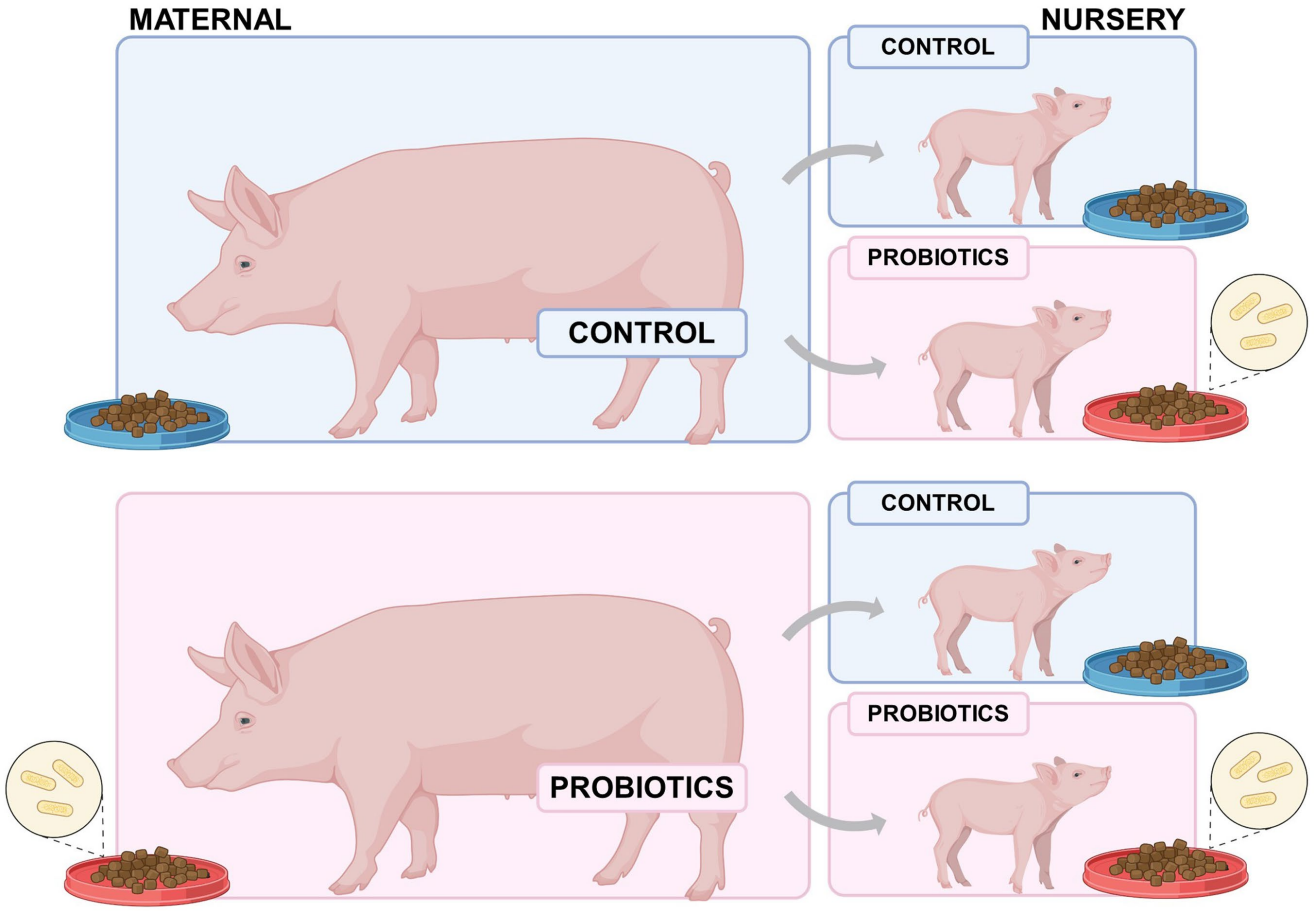
Experimental design of weaned born from probiotic-fed sows (maternal nutrition in gestation-lactation) or pigs fed the probiotic diet in the nursery (nursery nutrition).

Piglets were weaned at 21 days of age. From the entire group, 16 sows with parity order 4 (8 from the control group and 8 from the probiotic supplementation treatment) were selected. Two male piglets were selected per sow, considering those with individual weights closer to the modal weight of the litter at weaning. One of these piglets was assigned to the control group in the nursery phase and the other for the supplemented group in the nursery phase (Figure 1). Using this procedure, 32 males were transferred to the nursery trial. Sows and/or piglets treated with antibiotics during gestation and/or lactation were not considered for the trial, as well the piglets with diarrhea during any day from birth to weaning.

### 2.3 Nursery phase

The experiment was carried out in the experimental pig house at the Federal University of Rio Grande of Sul, located in Porto Alegre, Rio Grande of Sul, Brazil. The 32 male pigs (6.20 kg ± 410 g; Landrace x Large white) were individually housed in metabolism crates (1.12 meters long × 0.60 meters wide x 1.92 meters high) and split into one of the four treatments with eight replicates immediately after weaning (21 days) (Figure 1). The trial lasted for 22 days, which corresponded to 15 days of adaptation and 7 days for sample collection. The experimental diets provided during the nursery phase were formulated for the minimum cost solution to meet or exceed the nutritional requirements recommended by the Brazilian Tables for Poultry and Swine ((10), Table 1). The same reference was used for ingredient composition, except for soybean meal and corn, in which the total energy and crude protein content were analyzed before formulation and later used to estimate the metabolizable energy and digestible amino acid levels (10). Water and mash form feed were provided *ad libitum* throughout the adaptation periods (intake adaptation period 800 g and the coefficient of variation 12.5%). During the collection period (on days 15–21), the pigs received feed according to metabolic body weight (2.6 × the estimated maintenance requirement) (11). Temperature, humidity, and air circulation were controlled to ensure comfortable environmental conditions for the pigs.

**Table 1:** Ingredient formulas and chemical composition of experimental feeds used in trial.

Ingredients, %	Gestation	Lactation	Nursery
Corn	81.20	67.00	54.29
Soybean meal 46% CP	15.02	28.10	25.00
Meat and bone meal 48% CP	–	–	5.00
Whey powder	–	–	5.00
Soybean protein isolate 80% CP	–	–	4.30
Soybean oil	–	1.42	2.51
Spray-dried plasma	–	–	2.50
Premix^1^	0.50	0.50	0.50
Limestone	1.63	1.10	0.42
L-Lysine	0.16	0.35	0.17
DL-Methionine	0.05	0.10	0.11
L-Threonine	0.10	0.12	0.08
L-Tryptophan	0.005	0.05	–
L-Valine	–	0.16	–
Salt	0.42	0.50	0.08
Phytase^2^	0.005	0.005	0.005
Carbohydrases^3^	–	0.005	–
Calculated composition			
Crude protein, %	13.43	18.53	24.08
Digestible lysine %	0.65	1.10	1.34
Metabolizable energy, kcal/kg	3,311	3,400	3,370
Total calcium, %	1.00	0.91	0.97
Total phosphorus, %	0.45	0.55	0.64
Available phosphorus, %	0.37	0.45	0.52

^1^Premix with vitamins and minerals nursery phase. Vitamin A, 12.800.000,0 UI/kg; vitamin B1, 1.800,00 mg/kg; vitamin B2, 11.40 g/kg; vitamin K3 6.600,00 mg/kg; vitamin B6, 3.600,00 mg/kg; vitamin B12 52.000,00 mcg/kg; vitamin D3, 2.650.00,00 UI/kg; vitamin E, 72.200,00 UI/kg; folic acid 600.00 mg/kg; niacin, 80.00 g/kg; biotin 200.00 mg/kg; pantothenic acid, 40.00 g/kg. Calcium 90 g/kg; Phosphorus 20 g/kg; Sodium 35 g/kg; Cu 1000 mg/kg; Fe 1000 mg/kg; I 20 mg/kg; Mn 500 mg; If 8 mg/kg; Zn 15 g/kg.

^2^Natuphos (BASF Corporation, São Paulo, Brazil).

^3^Rovabio Advance (Adisseo, São Paulo, Brazil).

### 2.4 Digestibility and metabolism

Metabolism crates are equipped with trays for the total collection of feces and a system for total urine collection. Feces and urine were collected twice a day (8:00 a.m. and 5:30 p.m.). The beginning and end of collection periods were defined using an indigestible marker (0.5% of ferric oxide) mixed in the diets. All samples were stored and kept in a freezer in bags identified by the experimental unit.

At the end of the experimental period, fecal and urine samples were thawed at room temperature, weighed, and homogenized. Samples from each pig were collected and lyophilized. Then, the samples of feed, feces, and urine were analyzed for dry matter (oven at 105°C), nitrogen (micro Kjeldahl method), and gross energy (calorimetric pump), following the procedures described by AOAC (12). Coefficients of digestibility (dry matter, protein, and energy) and metabolizability (protein and energy), as well as the apparent metabolizable energy values, were calculated from the obtained data according to the equation provided by Sakomura and Rostagno (13). The manure production was defined by the total volume of all urine and feces collected during the digestibility period.

### 2.5 Feed retention rate and fecal moisture content

The time spent on the consumption of feed with ferric oxide and the appearance of marked feces were registered at the beginning and end of the trial. The feces collected each day were weighed and homogenized. A sample corresponding to 10% of the total weight was retained to determine dry matter content (drying oven at 105°C for 8 h).

### 2.6 Serum biochemistry

On days 3 and 22, the blood sample was collected from all pigs through vena cava puncture in a vacutainer without anticoagulant. The samples were held in the thermal box with ice for 40 min before centrifugation. Samples were centrifuged at 3500 rpm for 10 min and the serum was separated, collected, and frozen (−20°C) for later biochemical analysis. The concentrations of total protein, albumin, cholesterol, glucose, and alanine aminotransferase were determined using commercial analytical kits (Wiener lab, São Paulo, Brazil) and a semi-automated biochemical analyzer (Bioplus 2000®). The globulin concentration was calculated as the difference between total protein and albumin.

### 2.7 Intestinal permeability

Intestinal permeability was assessed on day 22 post-weaning. Six pigs of average weight and without diarrhea per treatment were selected to receive oral administration of a non-absorbable, high molecular weight fluorescent molecule at a dosage of 1 mL per pig (FITC-Dextran, 3,000 a 4,000 kDa); blood sample was collected 6 hours after FITC-Dextran administration. Blood was collected in a vacutainer without anticoagulant held in the thermal box with ice for 40 min and later centrifuged to separate the serum. The fluorescence levels of diluted serum (1: 1 in phosphate-buffered saline-PBS) were measured at an excitation wavelength of 485 nm and an emission wavelength of 528 (Speedscan, Analytik Jena, Jena, Germany), and the concentration per ml of FITC-Dextran serum, 3,000 a 4,000 kDa was calculated based on a standard curve, according to the methodology adapted from Vicuña et al. (14).

### 2.8 Organ weight

At the end of the experiment, all pigs were weighed and euthanized after insensitization by electrical stunning following exsanguination with agreed animal welfare and euthanasia standards described in the CONCEA euthanasia practice guidelines (15). The heart, liver, intestine, lung, kidney, and spleen were weighed and expressed in relation to body weight.

### 2.9 Intestinal morphology, resistance to rupture, and tight junctions

Samples from the duodenum, jejunum, and ileum (4 cm distal to the stomach for the duodenum, mid jejunum, and 4 cm distal to the jejunum for the ileum) were collected and preserved in flasks with 10% formaldehyde solution. Histological slides were made and stained with hematoxylin and eosin. Histological images of the slides were captured using a Digital Microcamera (Electronic Eyepiece Camera Video), coupled to a biological trinocular microscope (model TNB-41 T-PL, OPTON). In the intestinal fragments, villus length and diameter, and crypt depth were determined according to the methodology described by Caruso and Demonte (16). More details of the methodology used were presented by Galli et al. (17).

Segments of jejunum and colon (4 samples per pig, around 5 cm length per segment) were collected randomly immediately after slaughter. These segments were used to assess the intestinal resistance to rupture using a dynamometer (ITFG6005, Instrutemp, São Paulo, Brazil) that provides the ideal force necessary to break the sample (18). The results were expressed as kgf/cm.

Approximately 100 mg of tissue (jejunum) to evaluate tight junction zonula occludens-1 and occludin was homogenized in TissueLyser (Qiagen, Hilden, Germany), and the total RNA was purified by extraction with TRI® Reagent (Sigma, São Paulo, Brazil) chloroform. The extracts were treated with turboDNaseI (Ambion) and the RNA was quantified with NanoDrop (Thermo Scientific, USA). After RNA extraction, cDNAs were synthesized using high-capacity cDNA Synthesis cDNA Reverse Transcription (Applied Biosystems, Foster City, CA, USA), using 1 μg of RNA per reaction. The RT-qPCR reaction was performed by diluting the cDNAs in sterile MilliQ water (5x), and the targets were quantified using Bright-Green PCR Master Mix (Biotium, Fremont, CA, USA) in a QuantStudio 3 thermocycler. The cycling used was 95°C by 10 min, followed by 40 cycles of 95°C by 15 s and 60°C by 1 min. The Primer Express 3.0 program was used to design the oligonucleotide primers (Primer Sequences 5′ - > 3′). The *GAPDH* and *ACTB* genes were used as internal control.

In this type of analysis, each combination of target and sample generates a threshold value, Ct (threshold cycle), a relative measure of the concentration of target-specific messenger RNA (mRNA) in the sample. This value was normalized in the function of the expression of some reference gene, in this case, the *GAPDH* gene was used, generating a value of DCt (target Ct/Ct *GAPHD*). In addition to this normalization, the data was also normalized about the mean of the DCt value of the control group, generating DDCt value (DCt/mean DCt Control). *Zonula occludens-1* and *occludin* were expressed as mRNA relative expression.

Note code gene: ***Zonula occludens-1:*** Forward AAGCCCTAAGTTCAATCACAATCT; Reverse ATCAAACTCAGGAGGCGGC; ***Occludin:*** Forward TCCTGGGTGTGATGGTGTTC; Reverse CGTAGAGTCCAGTCACCGCA.

### 2.10 Statistical analysis

The individual (pig) was the experimental unit in all responses. All data were submitted to the Ryan-Joiner test to assess their normal distribution (Minitab, version 19). Analyses of variance were performed using PROC MIXED using (SAS, version 9.3). The fixed effects of pigs born from probiotic-fed sows or pigs fed the probiotic diet in the nursery and their interaction was considered. Serum biochemistry and fecal moisture content were analyzed as repeated measures over time. Means are presented in this report by treatments (main effect in pigs born from probiotic-fed sows or pigs fed the probiotic diet in the nursery) and the interactions were discriminated only when significant. Eventual differences were assessed with the Tukey multiple comparison test. The interpretation of the results was performed considering a 5% level as significant results and a 10% level as a trend.

## 3 Results

### 3.1 Gestation-lactation phase

Sow fed-probiotic had a higher number of piglets born alive compared to the control (*p* = 0.007). Sows fed-probiotic had a lower stillborn and mummified number in relation to the control (*p* < 0.05). Pigs born from probiotic-fed sows had higher birth weight, birth weight alive, daily weight gain, and weight at the weaning in relation to the control (*p* < 0.05; Table 2).

**Table 2:** Sow probiotic supplementation on litter performance.

Item	Control	Probiotic	RSE^1^	*p-*value^2^
Piglets born, n/litter	15.84	15.57	0.269	0.324
Piglets born alive, n/litter	14.05	14.66	0.226	0.007
Stillborn, %	1.899	1.553	0.101	0.041
Mummified, %	2.269	1.003	0.216	0.032
Birth weight, kg	1.237	1.307	29.75	0.007
Birth weight alive, kg	1.257	1.321	29.96	0.011
Daily weight gain, g/day	189.1	204.0	5.021	0.003
Weight at 21 days, kg	5,310	5,572	118.7	0.028

^1^RSE, residual standard error.

^2^*p*-value, Probability of treatment effect.

### 3.2 General results nursery phase

The average room ambient temperature was 25.1°C and the daily relative humidity averaged 78%. These values suggested that the pigs were housed under thermoneutral conditions. No pigs were removed from the experiment and no health issues were detected during the experimental period.

### 3.3 Feed digestibility and nutrients metabolism nursery phase

The interaction between probiotic supplementation for pigs born from probiotic-fed sows and pigs fed the probiotic diet in the nursery was tested for all variables. However, no significant interactions were observed in this study.

Pigs were fed allotment of the same amount provided per treatment during the collection period. The intake, however, was different, which had consequences on the nutrient intake due to the higher consumption (Table 3). Pigs fed the probiotic diet in the nursery did not affect pigs’ feed intake during the experimental period. However, an increase in feed intake was observed in pigs born from probiotic-fed sows compared to the control (*p* = 0.035).

**Table 3:** Feed variables of weaned pigs born from probiotic-fed sows during gestation-lactation (maternal nutrition) or pigs fed the probiotic diet in the nursery (nursery nutrition).

	Maternal nutrition		Nursery nutrition		RSE^1^	*p-*value^2^		
Item	Control	Probiotic	Control	Probiotic		MN	NN	MN × NN
Body weight (kg)	6.091	6.302	6.152	6.241	0.412	0.159	0.548	0.605
Feed allotment (g/day)	717.3	717.3	720.6	714.1	15.65	0.998	0.669	0.667
Intake (g/day)	585.1	611.8	596.7	600.3	12.07	0.035	0.762	0.957
Leftover (g/day)	132.2	105.5	123.9	113.8	22.15	0.096	0.510	0.697

^1^RSE, residual standard error.

^2^*p*-value, Probability of treatment effect for maternal nutrition (MN), nursery nutrition (NN), and interaction.

Pigs born from probiotic-fed sows had greater apparent total tract digestibility (ATTD) of dry matter (*p* = 0.046) and gross energy (*p* = 0.018, Table 4), as well as higher energy metabolizability (*p* = 0.023, Table 4). However, there was no difference in pigs fed the probiotic diet in the nursery of the same variables. There was no difference in the pigs born from probiotic-fed sows and pigs fed the probiotic diet in the nursery over protein digestibility, and metabolizability coefficients.

**Table 4:** Coefficients of digestibility and metabolizability of pigs born from probiotic-fed sows during gestation-lactation (maternal nutrition) or pigs fed the probiotic diet in the nursery (nursery nutrition).

	Maternal nutrition		Nursery nutrition		RSE^1^	*p-*value^2^		
Item	Control	Probiotic	Control	Probiotic		MN	NN	MN × NN
Dry matter (%)	88.63	89.56	89.32	88.87	0.447	0.046	0.310	0.865
Protein (%)	86.14	87.10	86.90	86.34	0.829	0.248	0.490	0.799
Energy (%)	87.34	88.46	88.20	87.60	0.441	0.018	0.176	0.487
Protein metabolizability (%)	84.65	85.40	85.20	84.85	0.754	0.323	0.629	0.907
Energy metabolizability (%)	84.60	85.73	85.19	85.14	0.471	0.023	0.915	0.416

^1^RSE, residual standard error.

^2^*p*-value, Probability for maternal nutrition (MN), nursery nutrition (NN), and interaction.

Pigs born from probiotic-fed sows also had higher nitrogen intake (*p* = 0.035), absorption (*p* = 0.007), and retention (*p* = 0.012, Table 5). This group also consumed more energy (*p* = 0.035), digestible energy (*p* = 0.017), and metabolizable energy (*p* = 0.023, Table 6). However, there was no difference in pigs fed the probiotic diet in the nursery over nitrogen and energy balance. Furthermore, no difference in feces, urine, or manure production was observed between treatments (Table 7).

**Table 5:** Nitrogen balance of weaned pigs born from probiotic-fed sows during gestation-lactation (maternal nutrition) or pigs fed the probiotic diet in the nursery (nursery nutrition).

	Maternal nutrition		Nursery nutrition		RSE^1^	*p-*value^2^		
Item	Control	Probiotic	Control	Probiotic		MN	NN	MN × NN
Intake (g/day)	22.12	23.13	22.56	22.69	0.456	0.035	0.760	0.958
Fecal (g/day)	3.065	2.953	2.941	3.078	0.212	0.594	0.512	0.783
Urinary (g/day)	0.322	0.384	0.369	0.337	0.060	0.374	0.644	0.500
Absorbed (g/day)	19.05	20.17	19.61	19.61	0.380	0.007	0.997	0.827
Retained (g/day)	18.73	19.79	19.24	19.27	0.391	0.012	0.942	0.933
Ratio ret./abs.^3^ (%)	98.28	98.04	98.05	98.27	0.367	0.510	0.534	0712

^1^RSE, residual standard error.

^2^P-value, Probability for maternal nutrition (MN), nursery nutrition (NN), and interaction.

^3^Ratio ret./abs., Ratio between protein retention and protein absorption.

**Table 6:** Energy balance of weaned pigs born from probiotic-fed sows during gestation-lactation (maternal nutrition) or pigs fed the probiotic diet in the nursery (nursery nutrition).

	Maternal nutrition		Nursery nutrition		RSE^1^	*p-*value^2^		
Item	Control	Probiotic	Control	Probiotic		MN	NN	MN × NN
Intake (kcal/day)	2,365	2,473	2,412	2,426	48.80	0.035	0.762	0.956
Fecal (kcal /day)	297.4	282.9	283.5	296.8	12.77	0.258	0.294	0.564
Urinary (kcal /day)	65.72	66.57	71.79	60.50	8.550	0.919	0.186	0.771
ED^3^ (kcal/kg)	3,530	3,576	3,565	3,541	17.93	0.017	0.175	0.485
EM^4^ (kcal/kg)	3,419	3,465	3,443	3,442	19.03	0.023	0.920	0.415
Ratio EM/ED (%)	96.86	96.90	96.58	97.18	0.980	0.913	0.152	0.828

^1^RSE, residual standard error.

^2^*p*-value, Probability for maternal nutrition (MN), nursery nutrition (NN), and interaction.

^3^ED, digestible energy.

^4^EM, metabolizable energy.

**Table 7:** Manure production (dry-matter basis) and feed retention rate of weaned pigs born from probiotic-fed sows during gestation-lactation (maternal nutrition) or pigs fed the probiotic diet in the nursery (nursery nutrition).

	Maternal nutrition		Nursery nutrition		RSE^1^	*p-*value^2^		
Item	Control	Probiotic	Control	Probiotic		MN	NN	MN × NN
Feces (g/day)	64.10	61.45	61.48	64.07	2.923	0.364	0.370	0.934
Urine (g/day)	31.49	31.94	32.24	31.19	4.080	0.911	0.793	0.964
Manure (g/day)	95.59	93.39	93.72	95.26	4.373	0.612	0.719	0.922
Feed retention rate (minutes)	1,391	1,156	1,276	1,272	457.8	0.028	0.705	0.962

^1^RSE, residual standard error.

^2^*p*-value, Probability for maternal nutrition (MN), nursery nutrition (NN), and interaction.

### 3.4 Feed retention rate and fecal moisture content

Pigs born from probiotic-fed sows had a faster feed retention rate (*p* = 0.028, Table 7), but pigs fed the probiotic diet in the nursery had no difference.

**Figure 2:**
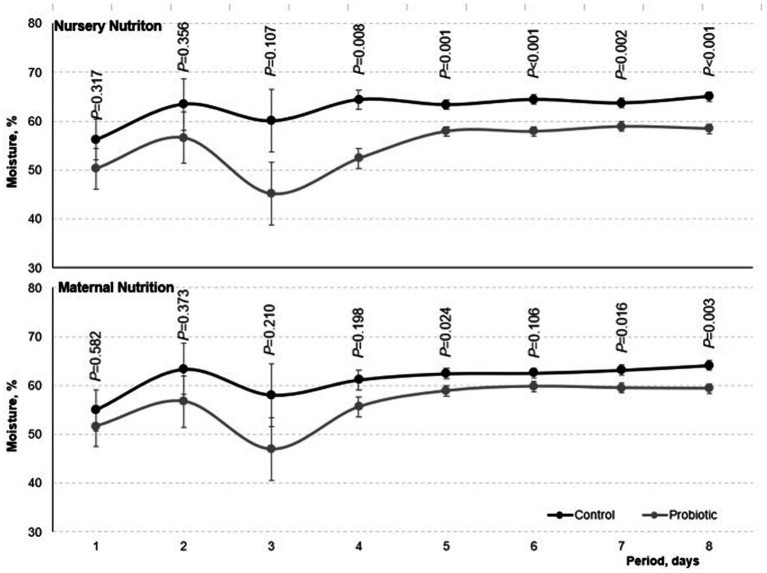
Fecal moisture of weaned born from probiotic-fed sows during gestation-lactation (maternal nutrition) or pigs fed the probiotic diet in the nursery (nursery nutrition). Probability of maternal nutrition and nursery nutrition  are presented in the figure. Effects of day (*p* <  0.05), interaction maternal nutrition by nursery nutrition (*p* >  0.05), maternal nutrition by day (*p* < 0.05), and nursery nutrition by day (*p* <  005) were also considered in the model. Period days 1–8 represent the digestibility period.

Pigs fed the probiotic diet in the nursery and pigs born from probiotic-fed sows reduced (*p* < 0.05) the fecal moisture of the nursery phase (Figure 2). Fecal moisture was lower in pigs fed the probiotic diet in the nursery compared to the control on the third day of the experiment (*p* < 0.10 on day 3, *p* < 0.05 on days 4,5,6,7, and 8). The same effect was observed in pigs born from probiotic-fed sows starting on the fifth day of evaluation (*p* = 0.106 on the sixth day, *p* < 0.05 on days 5, 7, and 8).

### 3.5 Intestinal permeability, morphology, resistance to rupture, tight junctions, and organ weight

Pigs born from probiotic-fed sows had reduced intestinal permeability (−16%; *p* = 0.034, Table 8) and increased jejunum breaking strength (+21%, *p* = 0.006, Table 8). Pigs fed the probiotic diet in the nursery showed a trend of reduced intestinal permeability (−10%; *p* = 0.094) and intestinal breaking resistance (+12%, *p* = 0.085). No difference in *zonula occludens-1* and *occludin* was observed in maternal and nursery nutrition.

**Table 8:** Intestinal permeability, tight junctions, gut resistance, and intestinal morphology of weaned of pigs born from probiotic-fed sows during gestation-lactation (maternal nutrition) or pigs fed the probiotic diet in the nursery (nursery nutrition).

	Maternal nutrition		Nursery nutrition		RSE^1^	*p-*value^2^		
Item	Control	Probiotic	Control	Probiotic		MN	NN	MN × NN
Dextran in serum (μg/mL)	0.405	0.339	0.393	0.350	0.040	0.038	0.088	0.458
*Zonula occludens-1^3^*	0.964	0.996	0.936	1.023	0.320	0.891	0.534	0.819
*Occludin* ^3^	0.949	0.929	0.877	1.002	0.310	0.882	0.356	0.384
Jejunum (kgf/cm)	1.377	1.660	1.431	1.607	0.102	0.006	0.085	0.982
Colon (kgf/cm)	1.340	1.279	1.263	1.356	0.093	0.457	0.265	0.340
Villi height (μm)	401.8	406.7	407.5	401.0	7.126	0.481	0.349	0.117
Villi width (μm)	116.1	110.7	114.7	112.1	4.454	0.216	0.546	0.859
Villi area (μm^2^)	47,759	44,871	47,528	44,871	2,487	0.172	0.251	0.468
Crypt depth (μm)	271.2	270.7	270.5	271.4	6.045	0.937	0.891	0.203
Ratio Villi height: Crypt depth	1.531	1.627	1.545	1.613	0.034	0.036	0.136	0.181

^1^RSE, residual standard error.

^2^*p*-value, Probability for maternal nutrition (MN), nursery nutrition (NN), and interaction.

^3^Zonula occludens-1 and occluding were expressed as mRNA relative expression.

**Figure 3:**
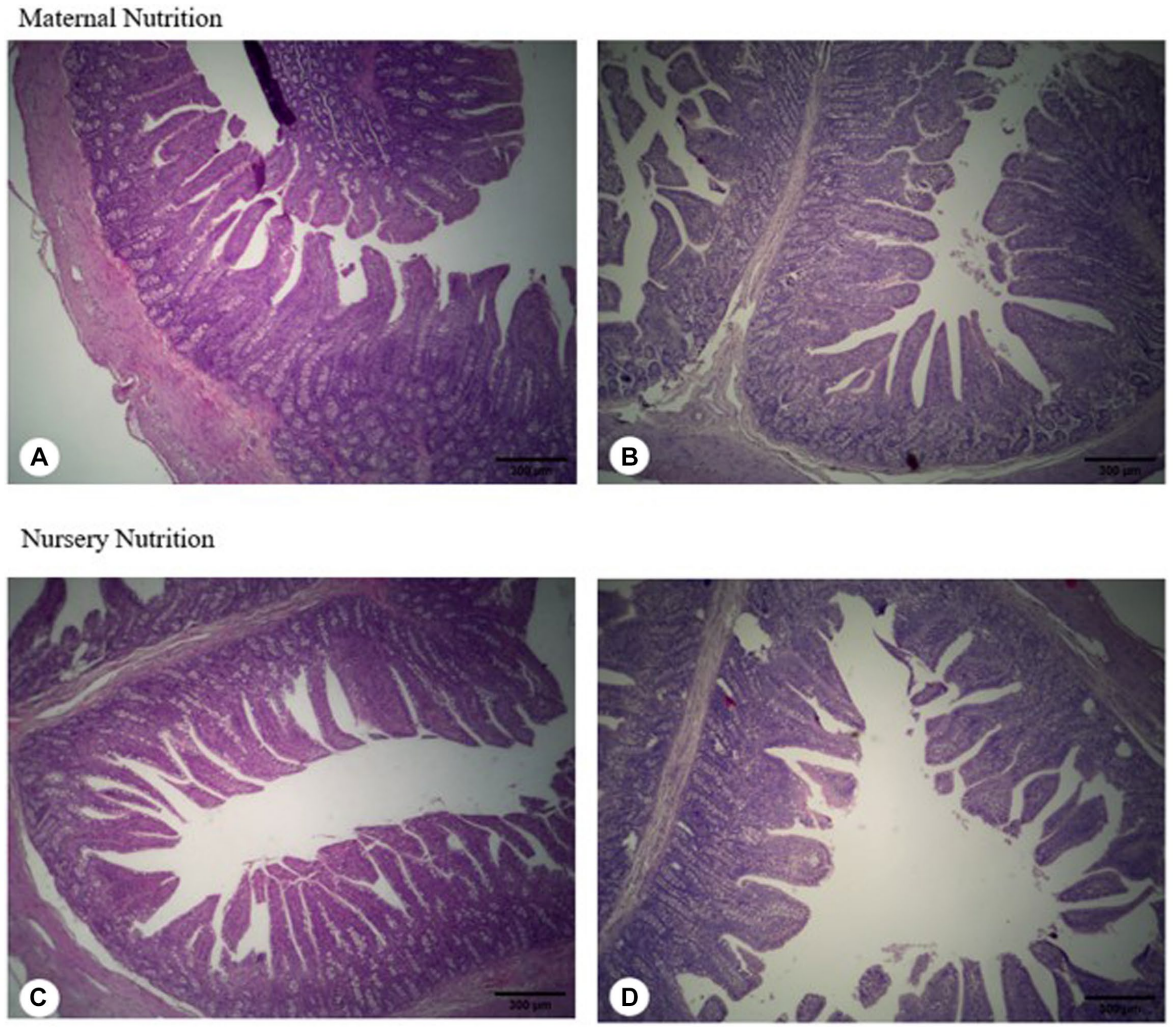
Jejunum morphology of weaned born from probiotic-fed sows during gestation-lactation (maternal nutrition) or pigs fed the probiotic diet in the nursery (nursery nutrition). **(A)** Pigs born from control-fed sows; **(B)** Pigs born from probiotic-fed sows; **(C)** Pigs fed the control diet in the nursery; **(D)** pigs fed the probiotic diet in the nursery.

There were minor differences in villus height, thickness, and area, as well as crypt depth (Table 8), where the villus: crypt ratio was greater (*p* = 0.036, Figure 3) in pigs born from probiotic-fed sows. For the maternal and nursery nutrition treatments, there was no difference in the relative weights of the organs (Table 9).

**Table 9:** Relative organ weight of weaned pigs born from probiotic-fed sows during gestation-lactation (maternal nutrition) or pigs fed the probiotic diet in the nursery (nursery nutrition).

	Maternal nutrition		Nursery nutrition		RSE^1^	*p-*value^2^		
Item	Control	Probiotic	Control	Probiotic		MN	NN	MN × NN
Intestine (%)	11.84	11.26	12.00	11.10	1.900	0.437	0.111	0.964
Heart (%)	0.602	0.622	0.614	0.610	0.082	0.570	0.884	0.160
Kidneys (%)	0.662	0.655	0.674	0.643	0.081	0.834	0.301	0.675
Spleen (%)	0.217	0.272	0.246	0.244	0.058	0.218	0.921	0.499
Liver (%)	2.930	3.110	3.090	2.960	0.310	0.163	0.246	0.366
Lungs (%)	1.220	1.360	1.290	1.290	0.150	0.130	0.992	0.200

^1^RSE, residual standard error.

^2^*p*-value, Probability for maternal nutrition (MN), nursery nutrition (NN), and interaction.

### 3.6 Serum biochemistry

Pigs born from probiotic-fed sows had greater cholesterol levels than the control (*p* = 0.047, Table 10). This same group showed reduced ALT enzyme levels compared to the control (*p* = 0.043).

**Table 10:** Serum biochemistry of weaned pigs born from probiotic-fed sows during gestation-lactation (maternal nutrition) or pigs fed the probiotic diet in the nursery (nursery nutrition).

	Maternal nutrition		Nursery nutrition		Days		RSE^1^	*p-*value^2^			
Item	Control	Probiotic	Control	Probiotic	3rd	22nd		MN	NN	MN × NN	Days
TP^3^ (g/dL)	6.456	6.018	6.340	6.134	6.878	5.596	0.413	0.115	0.450	0.598	0.004
Albumin (g/dL)	4.263	4.253	4.356	4.160	4.582	3.934	0.334	0.944	0.421	0.436	0.011
Globulin (g/dL)	2.356	2.031	2.166	2.220	2.584	1.803	0.189	0.243	0.845	0.834	0.023
CHOL^4^ (mg/dL)	79.74	96.78	89.30	87.22	135.3	41.21	6.280	0.047	0.801	0.868	<0.001
Glucose (mg/dL)	212.5	223.7	220.5	215.7	204.3	231.9	12.43	0.410	0.720	0.982	0.010
ALT^5^ (U/L)	54.77	47.78	51.06	51.49	58.49	44.06	2.321	0.043	0.896	0.266	<0.001

^1^RSE, residual standard error.

^2^*p*-value, Probability for maternal nutrition (MN), nursery nutrition (NN), interaction, and days.

^3^TP, Total protein.

^4^CHOL, Cholesterol.

^5^ALT, Alanine aminotransferase.

On day 3 of the nursery period, the levels of albumin, globulin, cholesterol, and ALT were higher (*p* < 0.05, Table 9) than on day 22 of the nursery period. Between day 3 and day 22, glucose levels tended to rise (*p* = 0.010). For cholesterol (*p* = 0.023) and glucose (*p* = 0.016) levels, there was an interaction between maternal supplementation and time of evaluation. In both cases, cholesterol (117.32 vs. 153.31 mg/dL) and glucose (185.76 vs. 222.87 mg/dL) were greater (*p* < 0.001; *p* = 0.010) on day 3 in pigs born from probiotic-fed sows than pigs from control and not different between pig groups on day 22.

## 4 Discussion

Probiotics are live microorganisms that help maintain digestive system health by improving gut microbiota (19). These effects may promote digestibility and improve performance. In this research, it was demonstrated that probiotic supplementation administered to sows during gestation and lactation improved metabolism and gut health but in the offspring even after weaning. While some positive effects were also observed when probiotics were directly supplied to weaned pigs, the majority of responses were more favorable when the supplementation was provided maternally.

The maternal influence on the development of piglets is widely acknowledged. The close contact between newborn piglets and their mothers also plays a crucial role in shaping the bacterial colonization of the porcine gastrointestinal tract in the early stages (8). This early colonization may have a lasting impact on the piglets, often referred to as “microbial imprinting.” Even so, studies that span multiple production phases, such as the period between gestation and maternity, as well as the nursery phase, can be challenging to carry out. However, such investigations are essential because these studies allow for the detection of management-related effects that may manifest at various stages of the production cycle. Thus, while these studies may present logistical difficulties, they are crucial for gaining a comprehensive understanding of the factors that influence animal husbandry practices. To the best of our understanding, this study represents the initial effort to assess the carryover impact of multi-species probiotics from the gestation and lactation periods on the nutrient digestibility and intestinal health of piglets during the nursery phase.

Zhang et al. (20) observed that sows supplemented with 0.2% *Bacillus subtilis* after 90 days of gestation had an increased number of live births, which is in agreement to the present study. However, most studies available in the literature have supplemented sows with probiotics after 30 days of gestation, making it difficult to claim that it is an effect of the probiotic on some performance responses (20–29) because some of these litter performances are determined in earlier stages of pregnancy. It is also important to highlight that the supplementation during the entire gestation period is more feasible in some industrial scenarios in which sows are usually provided with a single diet throughout the gestation phase.

Probiotic supplementation also may alter colostrum composition, which may be related to improved nutrient digestion and absorption in sows (21). Tsukahara et al. (22) found that sows probiotic fed with *Bacillus mesentericus*, *Clostridium butyricum*, and *Enterococcus faecalis* increased the concentration of IgG in milk and colostrum as a result, the litter showed greater weight gain compared to the control group. These facts could explain the increased daily weight gain and weight at weaning in pigs born to probiotic-fed sows. Hence, probiotic supplementation inhibits the proliferation of harmful bacteria and promotes beneficial microbial growth in sows (19), which can modulate the intestinal microbiota of the offspring, thereby improving gut health and nutrient and energy digestibility.

Evaluations of probiotic supplementation during the nursery stage are more common in the literature compared to maternal supplementation strategies. Indeed, several studies have demonstrated positive outcomes associated with probiotic supplementation. Lan et al. (23) found that the addition of a probiotic based on *Bacillus coagulans, Bacillus licheniformis, Bacillus subtilis,* and *Clostridium butyricum* to weaned pigs increased the digestibility of dry matter, protein, and crude energy. Furthermore, the use of probiotics can reduce pathogenic bacteria like *E. coli* while increasing the number of *Lactobacillus* in feces, reducing the competition for nutrients between pathogenic and beneficial microbiota (23). Yan et al. (24) found that *Lactobacillus planetarium* supplementation for weaned pigs increased the apparent digestibility of total tract, nitrogen, and gross energy. Lan et al. (25) also noted that *L. acidophilus* supplementation increased the digestibility of dry matter, protein, and crude energy in nursery pigs.

The current study has highlighted numerous beneficial outcomes, which can be ascribed to a multitude of variables. Several of these factors are exclusive to the specific microorganisms present in the probiotic utilized in the study. *L. acidophilus*, for example, is a microorganism that produces lactic acid, which reduces pH and inhibits the development of pathogenic microorganisms. Furthermore, the authors emphasize that *L. acidophilus* supplementation reduced *E. coli* counts while increasing *Lactobacillus* counts in feces, which justifies the improvement in digestibility parameters. Wang and Kim (26) found similar effects on *Lactobacillus* and *E. coli* counts, but with *L. plantarum* supplementation. In addition, *Lactobacillus* can produce digestive enzymes such as protease and phytase (27), which may explain the improved digestibility in the group that received probiotics. *Lactobacillus rhamnosus* can secrete antimicrobial compounds such as organic acids, hydrogen peroxide, or bacteriocins, which aid in pathogen inhibition and thus modulate the gut microbiota (28). The higher percentage of N absorbed and retained in pigs born from probiotic-fed sows indicates greater efficiency in utilizing the ingested and retained protein, which can be allocated for maintenance, muscle growth, and intestinal cell synthesis (29).

Lu et al. (30) tested the same experimental design of this trial (maternal vs. nursery supplementation) and observed that the supplementation of *Saccharomyces cerevisiae* increased de apparent total tract digestibility of dry matter, gross energy and phosphorus in pigs fed the supplemented diet in the nursery and for the pigs born from supplemented sows an increased gross energy and phosphorus. The authors suggested that supplementation with *Saccharomyces cerevisiae* was able to break down dietary fiber by microbiota modulation, which releases nutrients. It is possible that the observed improvement in digestibility among the probiotic group could be attributed to a similar action mechanism in this study.

One of the variables associated with the integrity and function of the intestinal mucosal barrier is gut morphology (31). Thus, the morphology of the intestinal mucosa is related to nutrient digestion and absorption, and thus, to animal growth (32). The villi:crypt ratio was higher in pigs from sows that received the probiotic, which helps to explain the increased digestibility in this group due to the increased surface area for nutrient absorption. Cai et al. (33) discovered that supplementing weaned pigs with *Bacillus subtilis* and *Bacillus amyloliquefaciens* increased villus height in the duodenum and jejunum. In addition, pigs born from probiotic-fed sows and pigs fed the probiotic diet in the nursery had a higher breaking force in the jejunum regardless of the supplementation period. This is beneficial because it indicates that the intestine is more resistant. In the case of infections, for example, the intestines sag and are easily torn due to the inflammatory process caused by pathogens, toxins, and other factors. Lower breaking force is expected in characterized by inflammatory conditions because the cell death in the gut epithelium as a response to chronic inflammation is one of the possible factors involved in this response (34).

Another indicator of gut epithelial barrier function is gut permeability (32). As a result, this barrier is controlled by a system composed of epithelial junction complexes known as junction proteins (32). Hence, for pigs born from probiotic-fed sows, the lower intestinal permeability was 16.29%, and for the pigs fed the probiotic diet in the nursery, it was 10.94% compared to the control groups is advantageous because it reflects a lower passage of toxins and pathogens into the intestinal lumen. This also explains why nutrient digestibility has increased. Lan and Kim (35) observed that supplementing sows with *Enterococcus faecium* reduced the diarrhea score of post-weaning pigs. Furthermore, the authors reported that supplementing sows with *E. faecium* increased the counts of *Lactobacillus* and *Enterococci* in pigs’ feces while decreasing the counts of *E. coli*. Kang et al. (36) reported that *Lactobacillus rhamnosus* supplementation reduced fecal scores and attenuated pro-inflammatory cytokine responses in post-weaning pigs. In agreement with the authors above, we observed that pigs born from probiotic-fed sows and pigs fed the probiotic diet in the nursery decreased feces moisture could be linked to fewer pathogenic bacteria entering the intestinal lumen.

Animal nutrition and metabolism, in addition to the functions of various tissues and organs, are partially reflected in serum biochemical responses (37). Serum ALT increases at hepatocyte damage. For this reason, its decrease in the group of pigs born from probiotic-fed sows is favorable, as it means less liver overload and even a protective effect. This result agrees with Zhu et al. (38) that the serum levels of ALT decreased in the sow and offspring pig fed with probiotics (*Lactobacillus plantarum* and *Saccharomyces cerevisiae*).

The increase in cholesterol levels in pigs born to probiotic-fed sows was unexpected. However, it may be related to the activity of the enzyme 3-hydroxy-3-methyl glutaryl-CoA reductase, a key enzyme in cholesterol synthesis, or it may be due to the modulation of the intestinal microbiota (39, 40). These are two possible explanations.

Based on the results of this study, the effects of probiotics on the digestibility of nutrients and intestinal health of nursery pigs can be indirect when the pigs are born from sows that have been fed diets with probiotics during gestation and lactation, or direct when the pigs themselves are fed probiotic in the nursery diets. Results also suggest that supplementing sows with probiotics during the gestational and lactational phases may be a more effective strategy than supplementing pigs in the nursery phase. Conducting studies that involve multiple production phases can be challenging. Nonetheless, the results observed in this study highlight the importance of such investigations as the piglet metabolism during nursery is highly associated to the previous phases in its life.

## 5 Conclusion

Probiotic supplementation for gestation-lactation sows’ benefits pigs in the nursery phase more than simply supplementing pigs at this stage. Pigs born from probiotic-fed sows had higher energy digestibility coefficients and absorbed nitrogen as well as improved intestinal health by lowering intestinal permeability and moisture, increasing villi:crypt ratio, and resistance to small intestinal breakdown. As a matter of fact, probiotics reduced the ALT enzyme, which indicates liver damage. However, pigs fed the probiotic diet just in the nursery improved intestinal health due to the decrease in intestinal permeability and the resistance increase of the jejunum, as well as decreasing fecal moisture. Therefore, there seems to be a long-term influence of sow probiotic supplementation on progeny through the nursery, compared to the nursery probiotic supplementation that warrants further investigation. Further investigation and exploration of this subject are deemed necessary and advisable for subsequent research endeavors.

## Data availability statement

The raw data supporting the conclusions of this article will be made available by the authors, without undue reservation.

## Ethics statement

The animal studies were approved by the institutional ethics committee on the use of animals (CEUA/UFRGS – Protocol 39604). The studies were conducted in accordance with the local legislation and institutional requirements. Written informed consent was obtained from the owners for the participation of their animals in this study.

## Author contributions

GG: Conceptualization, Data curation, Formal analysis, Investigation, Methodology, Writing – original draft, Writing – review & editing. IA: Conceptualization, Data curation, Methodology, Supervision, Writing – review & editing. CL: Data curation, Methodology, Visualization, Writing – review & editing. TS: Data curation, Methodology, Visualization, Writing – review & editing. CC: Data curation, Methodology, Visualization, Writing – review & editing. JP: Data curation, Methodology, Visualization, Writing – review & editing. GB: Data curation, Writing – review & editing. BS: Data curation, Writing – review & editing. CR: Data curation, Writing – review & editing. CF: Data curation, Writing – review & editing. MK: Conceptualization, Funding acquisition, Supervision, Writing – review & editing.
